# Clopidogrel and hip fractures, is it safe? A systematic review and meta-analysis

**DOI:** 10.1186/s12891-016-0988-9

**Published:** 2016-03-22

**Authors:** Christopher G. K. M. Soo, Paul K. Della Torre, Tristan J. Yolland, Michael A. Shatwell

**Affiliations:** Orthopaedic surgery department, Port Macquarie Base Hospital, Wrights Rd, Port Macquarie, NSW 2444 Australia; Orthopaedic surgery department, Concord Repatriation General Hospital, Hospital Rd, Concord, NSW 2139 Australia; Orthopaedic surgery department, John Hunter Hospital, Lookout Rd, New Lambton Heights, NSW 2305 Australia

**Keywords:** Orthopaedics, Clopidogrel, Anticoagulant, Surgical blood loss, Hip fracture

## Abstract

**Background:**

Femoral neck fractures in the elderly make up a large proportion of Orthopaedic surgical admissions each year. Operating on patients with clopidogrel poses a challenge because of the risk of bleeding and the difficulty deciding the optimal timing of surgery. The aim of this systematic review is to examine the published evidence to establish a set of guidelines for approaching neck of femur patients who are on clopidogrel.

**Methods:**

All comparative studies with an intervention group and a control group were considered. Data on patient blood transfusion exposures, units transfused, haemoglobin concentration and drop in haemoglobin were extracted and pooled using the fixed effects model. Heterogeneity of the intervention effect was assessed with the *I*^2^ statistic.

**Results:**

A total of 4219 studies were identified. After removal of duplicates and after exclusion criteria were applied, there were 14 studies to be included. All 14 were case series with controls. There was no significant heterogeneity amongst the studies. Pooled odds ratio for transfusion exposures was 1.24 (95 % confidence interval 0.91 to 1.71) however this was not statistically significant (*p* = 0.14). No significant mean differences were found for other primary outcome measures.

**Conclusions:**

On the available evidence, we recommend that these patients can be managed by normal protocols with early surgery. Operating early on patients on clopidogrel is safe and does not appear to confer any clinically significant bleeding risk. As reported in other studies, we believe clopidogrel, if possible, should not be withheld throughout the perioperative period due to increased risk of cardiovascular events associated with stopping clopidogrel. Care should be taken intraoperatively to minimise blood loss due to the increased potential for bleeding.

**Trial registration:**

This systematic review and meta-analysis has been registered on Research Registry on July 16, 2015. The Review Registry Unique Identifying Number is: reviewregistry61.

**Electronic supplementary material:**

The online version of this article (doi:10.1186/s12891-016-0988-9) contains supplementary material, which is available to authorized users.

## Background

Femoral neck fractures in the elderly make up a large proportion of Orthopaedic surgical admissions each year and the numbers worldwide are expected to reach 6.26 million cases a year by 2050 [1]. These patients often present with numerous co-morbidities including coronary artery disease, cerebrovascular disease and peripheral vascular disease. In fact, recent studies show both a high incidence of cardiovascular disease among operative hip fracture patients (63.3 %), as well as a higher risk of hip fractures among patients with cardiovascular disease [2–4]. Thus, anticoagulant therapy in patients presenting with hip fractures is becoming more and more prevalent.

Increasingly common is the use of clopidogrel, a thienopyridine derivative, which irreversibly binds to the platelet receptor adenosine diphosphonate (ADP) and thus inhibits platelet aggregation and thrombus formation [5]. The National Institute of Clinical Excellence (NICE) guidelines recommends clopidogrel for the prevention and treatment of occlusive vascular events in patients with recent stroke, myocardial infarction, acute coronary syndrome and established peripheral vascular disease [6]. This includes patients who have undergone percutaneous coronary intervention and coronary artery by-pass grafting. The half-life of clopidogrel is 8 h, but the affected platelets remain irreversibly inactivated and are replaced by new platelets after 5 to 7 days. Studies have shown patients to show a complete recovery of platelet function 7 days after the last clopidogrel dose [7].

Manufacturers and other published guidelines based on the physiological lifespan of the platelets recommend stopping clopidogrel at least 5-7 days before undergoing elective surgery to allow recovery of normal platelet function and avoid the perioperative risks of increased bleeding [8–11]. However there is no consensus regarding guidelines for the perioperative management of clopidogrel in patients with acute femoral neck fractures. In June 2009 the Scottish Intercollegiate Guidance Network (SIGN), published a national guideline for the management of hip fractures in elderly patients and recommended that surgery should not be delayed in patients receiving anti-platelet therapy (aspirin, clopidogrel or dipyridamole) [12].

The difficulty in managing these patients who are on clopidogrel exists because the increased risk of perioperative bleeding and higher risk of spinal haematoma [13] during the use of regional anaesthesia must be weighed against the risks of delayed surgery and the risks associated with the withdrawal of anti-platelet drugs. The difference between clopidogrel and many other anticoagulant medications is that there is no known method of reversing its antithrombotic effects acutely and the effectiveness of a fresh platelet transfusion in the event of excessive bleeding is controversial. There are in vitro and clinical studies that suggest platelet infusions are an effective method of reversing the effects of clopidogrel [14–16]. However more recently, there have been large-scale studies that have been unable to show any effectiveness of emergency platelet transfusions in patients on antiplatelet therapy [17–19].

The increased bleeding risk of clopidogrel in patients undergoing surgical procedures has been reported however there are limited reports to support this in orthopaedic literature. Most of the studies relate to cardiac surgery and in these studies clopidogrel has been reported to result in a four to five times increased risk of haemorrhage-induced surgical re-exploration and three times increased risk of blood transfusion post coronary artery bypass graft surgery [20–22]. Case reports of extensive retroperitoneal haematoma post lumbar sympathetic blockade and cervical epidural haematoma post epidural injection resulting in quadriparesis have further highlighted the bleeding risks associated with clopidogrel [23, 24].

To minimise the risks of perioperative bleeding related to clopidogrel, surgery can be delayed for at least 5 to 7 days. However the risks of delaying surgery in femoral neck fracture patients is well documented. Numerous studies have shown that a surgical delay in femoral neck fracture patients can lead to significantly poorer patient outcomes including an increased mortality rate, prolonged in-hospital stay time and a reduced rate of return to independent living [25–29]. Delay to surgery greater than 48 h has been shown to be independently associated with a higher mortality rate at 30 days and 1 year [30]. A prospective observational study of 2660 patients and found a significant increase in mortality in hip fracture patients delayed more than 4 days for surgery compared to those operated without delay (10.7 % vs 8.7 %) [31].

Another important consideration is that of the risk of withholding clopidogrel in these hip fracture patients with cardiovascular comorbidities. Studies have shown that fractures, surgical procedures and trauma induce both an inflammatory and coagulatory effect [32, 33]. Withholding clopidogrel in hip fracture patients can potentially induce a rebound effect and cause thromboembolic events whilst in this prothrombotic state. There are reports of a perioperative incidence of acute coronary syndrome of up to 20.2 % in patients with femoral neck fractures [34]. In patients who have had coronary stents inserted, cessation of clopidogrel treatment during the first year is associated with a 20 % risk of myocardial infarction and 45 % mortality rate [35]. This risk is of particular concern to those with drug-eluting stents, for which dual-antiplatelet therapy is prescribed on an empirical basis for 3-6 months after implantation, with life-long aspirin. Studies have shown that patients who prematurely cease clopidogrel therapy, have a significantly increased risk of hospitalisation and mortality within the first 11 months due to stent thrombosis [36]. In addition, there have also been rising concerns for patients with drug-eluting stents regarding the risk of late stent occlusion after cessation of clopidogrel [37]. This difficult balancing act in managing these patients has resulted in a lack of consensus about the best practice and safest approach, and this is demonstrated by a wide variation in policies between different Orthopaedic departments. A number of published surveys of orthopaedic departments across the UK and US have demonstrated this variation, each of them largely based on anecdotal evidence [38–41]. One survey of 139 UK orthopaedic departments published in 2007 revealed 41 % stopped clopidogrel and operated immediately, 19 % continued clopidogrel and operated immediately, 21 % stopped clopidogrel for at least 5 days preoperatively and 19 % had various alternative protocols [38].

The aim of the study was to determine if operating early on patients with neck of femur fractures who are on clopidogrel increases the risk of clinically significant bleeding, reflected in rate of blood transfusions and postoperative decreases in haemoglobin concentrations, when compared to patients who are not on clopidogrel. A secondary aim was to establish a framework for managing neck of femur patients who are on clopidogrel.

## Methods

### Search strategy

A comprehensive search was performed using the following databases: The Cochrane Library (Wiley, to February 2015), MEDLINE (Ovid, 1946 to February 2015) and EMBASE (Ovid, 1974 to February 2015), and Google scholar (to February 2015). The following keywords were used: Anticoagulant”, “Plavix”, “Clopidogrel”, “Thienopyridine”, “Antiplatelet therapy”, “Hip fractures”, “Femoral neck fractures”, “Neck of femur fractures”, “Orthopaedic surgery”, “Surgery”, “Bleeding”, “Blood loss” (Additional file 1). Additionally all references of the retrieved articles were also checked for additional relevant studies. Studies selected were original clinical studies that addressed the use of clopidogrel in patients undergoing surgery for neck of femur fractures. All comparative studies with a treatment group and a control group were considered. Data limits were set from all journals up to February 2015. Exclusion criteria were: (1) studies comparing non-clopidogrel anticoagulant medication, (2) studies without neck of femur patients, and (3) studies other than clinical studies such as reviews, letters, editorials and expert opinions.

### Data extraction

Data were extracted by one of the investigators (first author) and checked by a second investigator (second author). The authors were not blind to authorship, journal of publication, or results of the trials. Extracted data included assessment of study quality, study design, number of patients, patient characteristics (age, gender, fracture), surgical treatment, number of days free of clopidogrel, and perioperative use of other anticoagulants, and follow-up. Outcome measures were divided into primary and secondary. Primary outcome measures included (1) allogeneic and autologous blood transfusion exposures or average number of units transfused per patient and (2) a postoperative haemoglobin concentration or a drop in haemoglobin concentration. Secondary outcome measures were recorded from the included studies if available: average time to surgery, length of stay in hospital and postoperative complications (including haematoma, cardiovascular, cerebrovascular, thromboembolic events, death).

### Quality appraisal

The quality of the studies was appraised based on a select number of well described quality appraisal methods [42–44]. These were: (1) study design- whether the study met the requirements of our research question, for example a comparative study with clopidogrel patient groups and non clopidogrel patient groups; (2) prospective or retrospective study; (3) study population clearly specified and defined; (4) homogeneity concerning patient population- for example patients on clopidogrel only or patients who are also on aspirin; (5) transparency of outcome measures and assessment; (6) transparency of missing data; (7) appropriate data management and statistics in relation to our research question; (8) confounding variables assessed, measured and commented on- for example the concurrent use of aspirin. The same investigators scored the items and assessed bias, and any disagreements were resolved by consensus.

### Meta-analysis

This was done using the fixed effects model. Heterogeneity of the intervention effect was assessed with the *I*^2^ statistic. Data analysis was performed using Review Manager 5.1 (The Cochrane Collaboration, 2011).

This systematic review conforms to the Preferred Reporting Items for Systematic reviews and Meta-Analyses (PRISMA) standards (Additional file 2).

## Results

### Search results

**Fig. 1:**
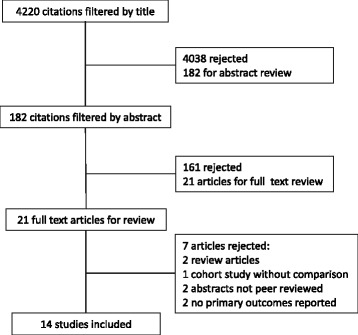
Flow diagram of literature search

The literature search identified 4220 possible eligible studies (Fig. 1) Initial screening of titles and removal of duplicates left 182 articles remaining. Exclusion criteria were studies comparing the use of other anticoagulant medication and surgical procedures not involving treatment of fracture of the femoral neck. The abstracts of these citations were reviewed and an additional 161 were rejected. This left 21 articles to be retrieved in full text and to be assessed for eligibility. 14 articles were included in the qualitative synthesis and 7 excluded. Of the 7 articles excluded: two were review articles [45, 46], one was a cohort study without a comparison [47], two were published abstracts from an international meeting and not published peer reviewed articles [48, 49], and two studies which included no primary outcome measures (only secondary outcome measure of complications) [50, 51].

### Study characteristics

All the reports were from a single centre except Chechick et al. [52] and Nydick et al. [53] who enrolled study patients from two orthopaedic trauma centres, and Feely et al. [54] which was a population based study using the records of health care providers in the Olmsted County, Minnesota (as part of the Rochester Epidemiology Project). The earliest study was from 2007, and the most recent was from 2014. The total number of patients across all the studies was 2938. The largest population size was 1225 patients, with 30 in the clopidogrel group (CG) and 1195 in the control or non-clopidogrel group (NCG) (Wordsworth et al. [55]). The study characteristics of all the included studies are summarized in table 1.

**Table 1:** Study characteristics and key results

Report	Type of study	Study size	Clopidogrel group- (n)	Control/Non-clopidogrel group- (n)	Key results
Harty et al. 2007 [68]	Case-control (Retrospective)	180	21	159	Higher mortality rate in CG than NCG (*p* = 0.003) CG were delayed to surgery compared to NCG (7.2 vs 2.1 days, *p* = 0.03)
Johansen et al. 2008 [56]	Case-control (Retrospective)	17	(<5 days) 7 (>5 days) 10		Less drop in Hb in the CG > 5 group (*p* = 0.01) Thromboembolic complications in delayed group (1 death from pulmonary embolus)
Sim and Gonski 2009 [57]	Case-control (Retrospective)	135	21 (<5 days) 16 (>5 days) 5	114	No significant difference in all outcomes for: CG vs NCG CG < 5 vs CG > 5
Cox et al 2009 [58]	Case-control (Retrospective)	20	(<7 days) 11 (>7 days) 9		Less drop in Hb in the CG >7 group (p < 0.05)
Nydick et al. 2010 [53]	Case-control (Retrospective)	50	(23	27	No significant difference in outcomes between CG and NCG
Chechik et al. 2011 [52]	Case-control (Prospective)	51	29	22	Significantly more blood loss in patients on Clopidogrel
Wallace et al. 2012 [63]	Case-control (Retrospective)	110	52	58	Statistically significant higher transfusion rates in CG (56 % CG vs 31 % NCG, *p* = 0.01)
Collinge et al. 2012 [62]	Case-control (Retrospective)	659	40	619	No significant difference in outcomes between CG and NCG
Chechik et al. 2012 [59]	Case-control (Retrospective)	60	(<5 days) 30 (>5 days) 30		No significant difference in outcomes between CG < 5 and CG >5
Hossain et al. 2013 [61]	Case-control (Retrospective)	102	50	52	No significant difference in outcomes between CG and NCG
Feely et al. 2013 [54]	Case-control (Retrospective)	120	40	80	No significant difference in outcomes between CG and NCG
Al Khudairy et al. 2013 [60]	Case-control (Retrospective)	47	(<5 days) 24 (>5 days) 23		No significant difference in outcomes between CG < 5 and CG >5 CG > 5 had 2 pre-op cardiac complications and 4 patients died from cardiac complications. CG < 5 had 2 patients die from cardiac complications.
Wordsworth et al. 2013 [55]	Case-control (Prospective)	1225	30	1195	No significant difference in outcomes between CG and NCG
Manaqibwala et al. 2014 [64]	Case-control (Retrospective)	162	15	147	No significant difference in time to outcomes between CG and NCG Significantly lower preop Hb and higher ASA in CG

All of the studies were comparative cohort studies, ten of which compared neck of femur fracture patients who were on clopidogrel (CG) with control patients who had never been on clopidogrel (NCG). Five studies performed comparative analysis of clopidogrel patients who had surgery within 5 or 7 days (CG < 5 or <7), and those who were on clopidogrel but had their surgery delayed for a minimum of 5 or 7 days (CG > 5 or >7) [56–60]. One study included both types of comparative analysis [57].

### Primary outcomes

Thirteen out of the fourteen studies included either the number of transfusion exposures or the average number of units transfused per patient. Twelve out of fourteen studies described postoperative haemoglobin or a drop in haemoglobin. The primary outcomes for the studies are summarized in the table 2.

**Table 2:** Primary outcome data

	CG transfusion exposures/total pt	NCG transfusion exposures/total pt	*P*-value	CG mean total units transfused per patient^a^	NCG mean total units transfused per patient^a^	*P*-value	CG postop Hb (g/dL)^a^	NCG postop Hb (g/dL)^a^	*P*-value	CG drop in Hb (g/dL)^a^	NCG drop in Hb (g/dL)^a^	*P*-value
Harty et al 2007 [68]							9.8	9.6	0.68			
Johansen et al 2008 [56]				1.0	0.2	-				3.7	2.4	0.01
Sim and Gonski 2009 [57] CG vs NG	4/21	37/114	0.30				10.7 (1.7)	10.3 (1.6)	0.44			
Sim and Gonski 2009 [57] CG <5 vs CG >5	2/16	2/5	>0.05				10.3	10.9	>0.05			
Cox et al 2009 [58]				0.9	0.2					3.1	1.8	<0.05
Nydick et al 2010 [53]	16/23	12/27	0.13							1.50	1.77	0.54
Chechik et al 2011 [52]				1.38 (0.98)	1.09 (1.38)	>0.05						
Wallace et al 2012 [63]	29/52	18/58	0.01				9 (1.9)	9.5 (1.7)	0.41			
Collinge et al 2012 [62]	22/40	342/619	0.89	1.4 (1.7)	1.5 (2.3)	0.50	10.1 (1.2)	10.2 (1.2)	0.52			
Chechik et al 2012 [59]	6/30	9/30	0.38	0.37	0.47	0.64				2.6	1.8	0.19
Hossain et al 2013 [61]	4/50	2/52	0.37	2.0 (0)	2.5 (0.7)	0.16	10.8 (1.5)	11.1 (1.5)	0.37			
Feely et al 2013 [54]	9/40	14/80	0.51	2.1 (2.1)	2.1 (3.5)	0.49	9.1 (1.2)	9.0 (1.1)	0.64	3.0 (1.7)	3.5 (1.6)	0.08
Al Khudairy et al 2013 [60]	6/24	8/23	>0.05	0.8 (1.6)	0.7 (1.1)	>0.05				1.5	1.1	>0.05
Wordsworth et al 2013 [55]	9/30	309/1195	0.67	0.67	0.55	0.54						
Manaqibwala et al 2014 [64]				0.9 (1.1)	0.6 (1.4)	0.23	10.3 (2.2)	11.4 (1.5)	0.07	0.8 (1.0)	0.6 (1.3)	0.32

^a^Values are mean (Standard Deviation)

Most studies presented good preoperative data to help assess the matching of the two groups. The most useful of these were patient age and sex, type of fracture and type of surgery, and ASA (American Society of Anesthesiologists) score. In terms of primary outcome data comparison, we felt the best guide was the postoperative Haemoglobin (Hb) or the drop in Hb, even though this in itself is an indirect measure of blood loss, influenced by several other patient and perioperative factors. This was unfortunately poorly recorded in some studies and often described at different time points e.g. immediately post op, day 1 post op, day 2 post op, at the point of discharge. Some studies described the lowest haemoglobin level during the postoperative period. The ideal point of time for the Hb level would be immediately postoperatively because this would usually be prior to blood transfusions (Except those given preoperatively or intraoperatively).

The other primary outcome measure we used was transfusion exposures and mean total units transfused per patient. Ten of the studies included data on both measures. However describing blood loss in terms of transfusion requirements is difficult due to the variability in transfusion protocols, reflected by the wide variation in transfusion rates between studies (from 5.8 % total exposures as reported by Hossain et al. [61] up to 55.3 % as reported by Collinge et al. [62]).

### Secondary outcomes

Secondary outcome measures were also recorded from the included studies if available. These were average time to surgery, length of stay in hospital and postoperative complications (including haematoma, cardiovascular, cerebrovascular, thromboembolic events and death). The secondary outcomes are summarised in table 3.

**Table 3:** Secondary outcome data

	Average time to theatre (days): CG/NCG	*P*-value	Length of hospital stay (days): CG/NCG	*P*-value	Complications					
					Haematoma/Haemorrhage (n)^a^: CG/NCG	*P*-value	Cardiovasc cerebrovasc/TE (n): CG/NCG	*P*-value	Death within 30 days (n):CG/NCG	*P*-value
Harty et al 2007 [68]	7.2/2.1	0.03	7.4/3.1	0.02					6/6	0.003
Johansen et al 2008 [56]	2.7/7.3						0/2			
Sim and Gonski 2009 [57] CG vs NG	3.5 (3.2)/0.9 (0.8)	<0.001	23.1 (17.7)/22.6 (21.4)	0.92	1/1				1/2	
Cox et al 2009 [58]			25/30	<0.05			2/0		2/1	
Nydick et al 2010 [53]	1.88/1.68	0.64			0/0		1/1		0/1	0.35
Chechik et al 2011 [52]	2.5 (1.5)/1.6 (0.96)	0.003			1/1		6/1		0/0	
Wallace et al 2012 [63]										
Collinge et al 2012 [62]			6.0 (3.4)/5.6 (4.0)	0.61	0/10	0.89	0/9	0.95	3/39	0.23
Chechik et al 2012 [59]	1.7 (1)/7.5 (2.71)	<0.001	11.1 (4.8)/17.7 (7.2)	0.0002	1/3	0.50	3/5	0.49	0/2	0.16
Hossain et al 2013 [61]			17.3 (13.3)/20.5 (16.6)	0.28	3/1	0.36			0/0	
Feely et al 2013 [54]	1.1 (0.7)/1.3 (1.3)	0.77			2/3	1.00	10/10	0.28	11/23	0.23
Al Khudairy et al 2013 [60]	4.2 (1)/8 (1)	<0.05	21.2 (11.9)/28.7 (16.4)	>0.05	0/0		0/2		2/4	
Wordsworth et al 2013 [55]	1.2/1.2	0.91			0/2	1.00			2/74	0.71
Manaqibwala et al 2014 [64]	2.3 (2.0)/1.9 (2.9)	0.25	10.6 (5.6)/7.4 (7.6)	0.04	1/2	0.25	2/6	1.00	1/6	0.24

Values are mean (Standard Deviation)

^a^Haemorrhage includes serious bleeding such as GI Bleed or Intracranial Haemorrhage

The most consistently reported secondary outcome measures were time from admission to theatre and mortality rate, with eleven out of the fourteen studies having reported both of these. Average time to theatre was a good outcome measure to help assess the effect of time to surgery on primary outcomes, and also to help assess for confounding. Five studies [56–60] specifically grouped their study patients according to time to surgery as their primary aim. Out of the remaining nine studies that did not separate the groups in terms of time to surgery, seven of them had a standard protocol of operating on all patients (including the patients on clopidogrel) as soon as possible without delay. This helps reduce the possible confounding factor of trying to compare the Clopidogrel group and control group with different times from admission to theatre.

Other complications and length of hospital stay were less consistently reported. Average length of hospital stay showed a wide variation, most likely reflecting the differences in hospital protocols or health care systems in different regions and countries.

### Meta-analysis

**Fig. 2:**
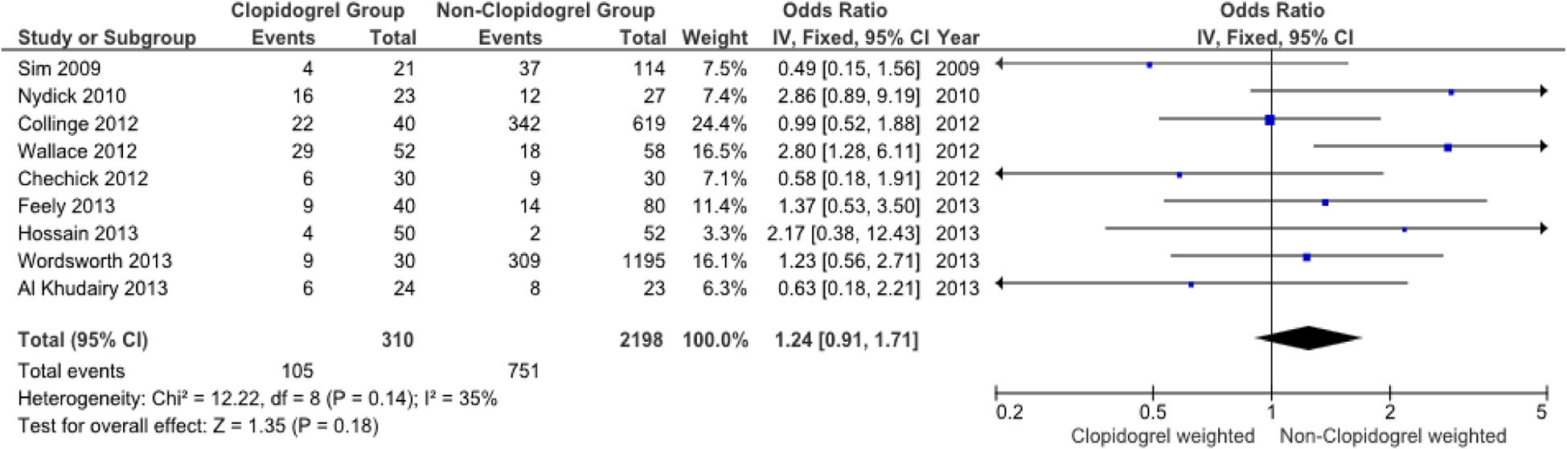
Meta-analysis of clopidogrel group and non-clopidogrel group: transfusions exposures

**Fig. 3:**
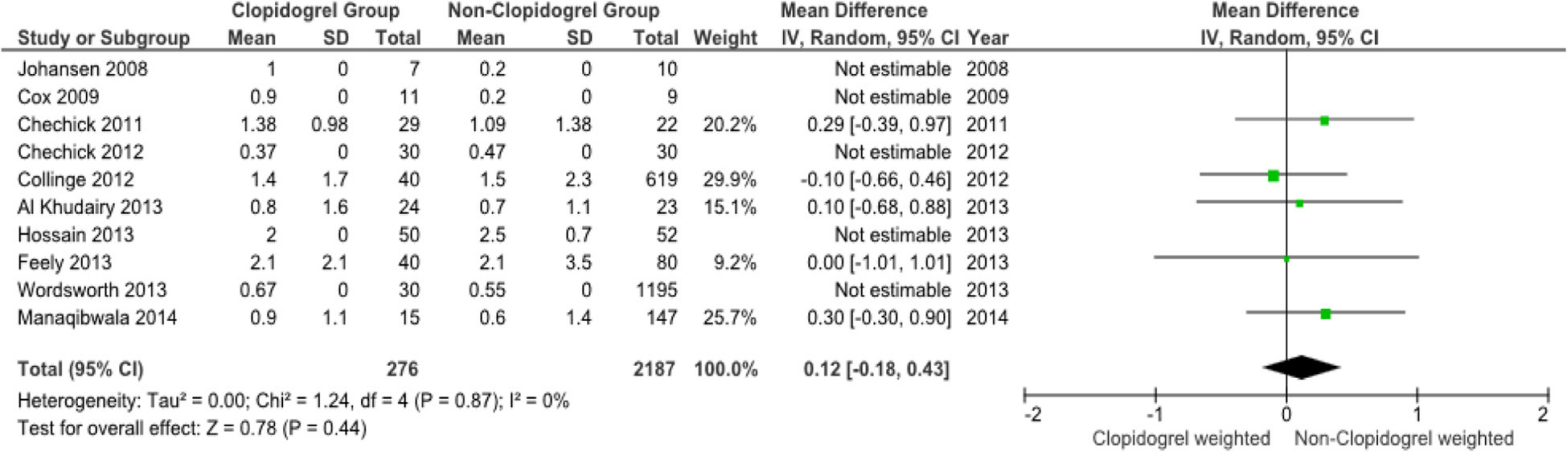
Meta-analysis of clopidogrel group and non-clopidogrel group: total units transfused per patient

**Fig. 4:**
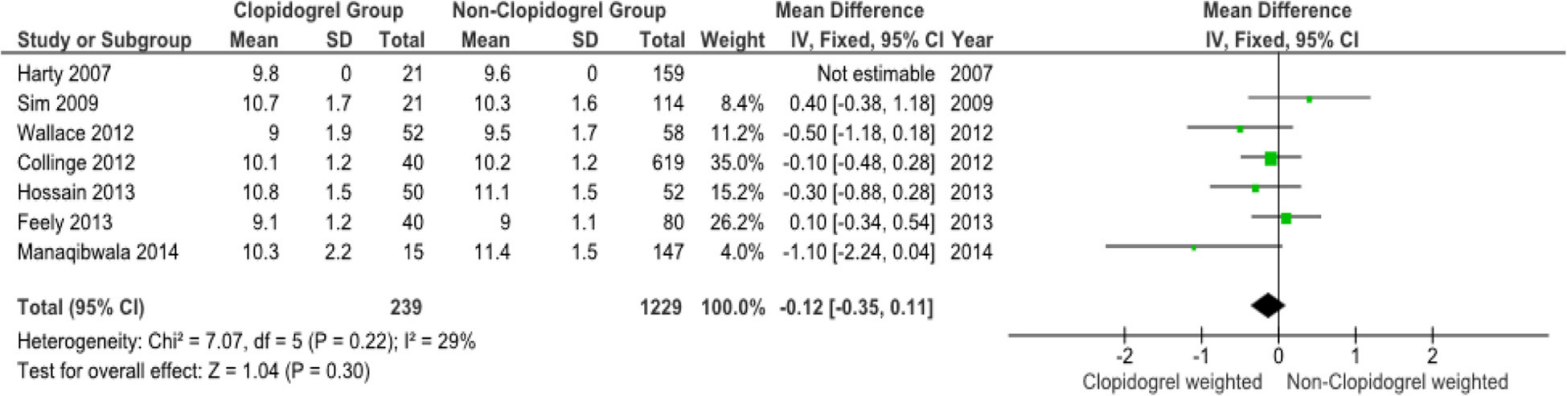
Meta-analysis of clopidogrel group and non-clopidogrel group: postoperative haemoglobin

**Fig. 5:**
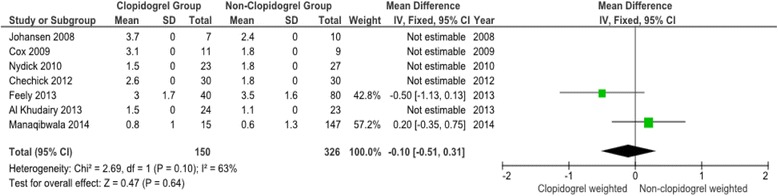
Meta-analysis of clopidogrel group and non-clopidogrel group: drop in haemoglobin

Meta-analysis was performed on all primary outcome measures. Forest plots are presented for each measure (Figs. 2, 3, 4 and 5). The forest plot for transfusion exposures (TE) (Fig. 2) shows the Odds Ratio (OR) for the relevant studies and we found no evidence of significant heterogeneity between the trials (Heterogeneity: Chi^2^ = 12.22, df = 8 (*P* = 0.14); I^2^ = 35 %). The pooled OR for the nine studies that reported TE was 1.24 (95 % confidence interval 0.91 to 1.71) with weighting towards increased transfusion exposures in the control group however this was not statistically significant (*p* = 0.18).

The forest plots for the remaining three primary outcome measures, total units transfused per patient (TUT), post-operative haemoglobin (POH) and drop in haemoglobin (DH) (Figs. 3, 4 and 5) show minimal mean differences between clopidogrel and control groups. Further, any significant mean difference was found to be not statistically significant: TUT mean difference 0.12 (95 % CI -018 to 0.43, *p* = 0.44), POH mean difference -0.12 (95 % CI -0.35 to 0.11, *p* = 0.30), DH mean difference -0.10 (95 % CI -0.51 to 0.31, *p* = 0.64). No significant heterogeneity being found between studies for each outcome.

## Discussion

This systematic review and meta-analysis of the 14 comparative studies provides evidence that operating early on neck of femur patients who are on clopidogrel is safe and poses no increased risk of bleeding when compared to patients not on clopidogrel.

In review of the literature, there is a paucity of high-level evidence to guide the perioperative management of patients with neck of femur fractures who are also on clopidogrel. Most of the literature is retrospective and non-randomised, and this leads to weak conclusions. Out of all the literature, only fourteen of the available studies are comparative, none of which are RCTs, and only two of which is prospective. Meta-analyses of observational studies also possess limitations due to the inherent biases that exist in nonrandomized, unblinded studies. The difficulty in creating large scale randomised clinical trials lies in the complexity of the patient with femur fractures and their individual and variable medical comorbidities.

### Search strategy

The electronic search strategy proved to be effective, generating all 13 of the 14 included studies. Only one of the included studies was not generated from the electronic searches, and this was from a reference search of an included study.

### Primary outcomes

The assessment and analysis of the data for the primary outcome measures was difficult due to the studies presenting their outcomes in different formats.

Most of the studies (13 out of 14) had good patient demographic data to assess whether the CG and control group were similar. The most useful was assessment of age, ASA score, type of surgery and type of fracture, and most of the studies had matched groups. Only one study reported a significant difference in age between the two groups [63]. As expected most studies showed a significantly higher number of either cardiovascular or cerebrovascular disease comorbidities in the intervention group, however surprisingly only two studies showed a significant difference in ASA grade [62, 64]. One study reported a significantly higher number of premorbid cerebrovascular accidents and transient ischaemic attacks in CG potentially introducing bias for providing blood transfusions (doctors may have a lower threshold to transfuse these patients). Of the four studies reporting the type of anaesthesia used during surgery, there was no statistically significant differences between the two groups [54, 55, 58, 61]. Despite the potential risk of spinal haematoma in patients using clopidogrel, no anaesthetic complications were reported in any of the four studies.

In comparing the primary outcomes between the CG and the controls, the best guide to assess blood loss and blood replacement is the postoperative Hb. However this was poorly recorded in some studies, and it was also reported at different times postoperatively e.g. immediately post op, 24 h post op, 48 h post op, at point of discharge. Some also only documented the lowest Hb recorded during the postoperative period. Ideally it should be taken at point of discharge because some patients had blood transfusions after the operation and before discharge.

The drop in Hb was poorly reported but the number of transfusion exposures and the mean units transfused per patient were generally well reported.

In the studies that reported on transfusion exposures, and mean units transfused per patient, most found no statistically significant difference between the groups. Only one study showed a significant increase in transfusion exposures in the CG [63]. In terms of Hb concentrations, there were two studies that reported a significantly higher drop in Hb in the CG [56, 58]. One study described a significant increase in perioperative blood loss (which was not part of our outcome measures due to rarity and inconsistency of reporting), however they found no significant difference in transfusion rates [52]. Thus there appears to be good evidence to suggest clopidogrel use does not increase transfusion rates or have significantly more effect on Hb falls.

A potential confounding factor in the results may be the concurrent use of aspirin in these patients. Dual-antiplatelet therapy is the mainstay of postoperative management of cardiac stent patients. Aspirin use has been described in a number of studies to be a risk factor for increased blood loss and transfusion requirements in hip fractures and hip fracture surgery [65–67].

In our report 8 out of the 14 studies reported on aspirin use. Only one of these studies showed a significant increase in aspirin use in the CG compared to controls and this study reported no significant difference in transfusion rates or Hb concentrations between the groups [55]. Only one study reported on blood loss in patients on clopidogrel as well as in patients on clopidogrel and aspirin combined [52]. Their results showed a significant increase in perioperative blood loss in both these groups when compared to patients not on any antiplatelet therapy, however there was no difference in transfusion rates or Hb concentrations.

### Secondary outcomes

The time to theatre was a well reported in most studies and it provided data not only comparing CG and controls but also on the effect of early surgery versus delayed surgery in patients on clopidogrel. Four studies assessed the differences in outcomes between early and delayed surgery in CG patients and none of these studies found a significant difference in bleeding outcomes. However three of these studies reported increased complications in the delayed surgery group such as pulmonary emboli, cardiovascular complications and decubitus ulcers. The results of these studies suggests that it is unnecessary to delay surgery for patients on clopidogrel, and on the contrary, delaying surgery may in fact pose risks of serious complications. One of the significant concerns for patients on clopidogrel is the implications of ceasing the antiplatelet therapy. It is well reported that discontinuation of antiplatelet therapy in patients with stents (especially drug-eluting) significantly increases the risks of cardiac stent thrombosis and death. In correlation with the results of our study, which suggest an increase in cardiovascular complications relating to the with-holding of clopidogrel prior to surgery, we recommend that patients with stents should continue clopidogrel or at very least require special consideration and discussion with the treating cardiologist about continuing or ceasing anti-platelet therapy.

Seven studies provided data on length of stay and this varied widely between studies. This probably represented differences in health care systems and policies in different areas and countries. Four of these studies found that being on clopidogrel significantly prolongs the length of stay in hospital but it is important to note that two of them had significant delays to surgery.

The reporting and statistical analysis of other adverse outcomes such as haematoma, haemorrhage, cardiovascular episodes and mortality was variable between studies. There were no significant differences in adverse outcomes between CG and controls, except in one study which showed a significantly increased mortality rate in the CG [68].

This study’s results differ slightly with a recently published systematic review and meta-analysis by Doleman and Moppett [69] which found that in the seven studies they included in their meta-analysis, there was an overall increase in the proportion of patients receiving blood transfusions in the clopidogrel group when compared to the control group (*p* = 0.05). This difference is accounted for by the different studies included in the meta-analyses (nine studies included in our meta-analysis). However the authors found no difference in mean units transfused and stated that the under-powering of the included studies prevented detection of any differences in postoperative complications.

## Conclusions

The combination of increasing population age, the increasing use of clopidogrel and the increasing number of hip fracture hospital admissions creates an important issue and potential management concern for orthopaedic surgeons. There is a relatively large collection of low-quality evidence showing operating early on neck of femur fracture patients on clopidogrel to be safe. However there is still wide variation in clinical practice and no consensus on recommendations.

The aim of this paper was to gather the best evidence available on the effect of clopidogrel on hip fracture surgery patients. Most of the literature is made up of low quality retrospective non-randomised cohort studies with small sample sizes. Ultimately, large multi-centred, adequately powered, well-designed randomised trials are needed to establish clearer guidelines for the management of these patients.

On the available evidence, we recommend that these patients can be managed by normal protocols with early surgery. Operating early on patients on clopidogrel is safe and does not appear to confer any clinically significant bleeding risk. As reported in other studies, we believe clopidogrel, if possible, should not be withheld throughout the perioperative period due to an increased risk of cardiovascular events associated with stopping clopidogrel. Care should be taken intraoperatively to minimise blood loss due to the increased potential for bleeding.

### Ethics approval and consent to participate

Not applicable.

### Consent for publication

Not applicable.

### Availability of data and materials

Data available from published papers as per references.

## Additional files

Additional file 1: Example OvidSP search terms for medline database. (DOCX 15 kb) Additional file 2: PRISMA 2009 checklist. (DOC 63 kb)

## Abbreviations

CG: Clopidogrel group
NCG: Non-clopidogrel group
Hb: Haemoglobin
ASA: American society of anesthesiologists
TUT: Total units transfused per patient
POH: Post-operative haemoglobin
DH: Drop in haemoglobin
