# Supplementary material for: Clopidogrel and hip fractures, is it safe? A systematic review and meta-analysis
Source: BMC Musculoskelet Disord. 2016 Mar 22;17:136. doi: 10.1186/s12891-016-0988-9 (PMC4804516; doi:10.1186/s12891-016-0988-9)
Supplement: Additional file 1: — Example OvidSP search terms for medline database. (DOCX 15 kb) [file 12891_2016_988_MOESM1_ESM.docx]

| 1. (randomized controlled trial or controlled clinical trial).pt. 2. exp Evidence-Based Medicine/ 3. exp Meta-Analysis/ 4. (systematic adj review).mp 5. or/1-4 6. exp surgery/ 7. exp Orthopedics/ 8. Orthop?edic$.tw 9. 6 and 7 and 8 10. exp Anticoagulants/ 11. Anticoagul$.tw 12. exp Ticlopidine/ 13. Ticlopidine.tw 14. exp Clopidogrel/ 15. Clopidogrel.tw 16. Plavix.tw 17. exp Platelet Aggregation Inhibitors/ 18. or/10-17 19. exp Femoral Neck Fractures/ 20. exp Hip Fractures/ 21. or/19-20 22. exp Hemorrhage/ 23. H?emorrhage.tw 24. exp Blood Transfusion/ 25. or/22-24 26. 5 and 9 and 18 and 21 and 25 |
| --- |

**Supplementary 1. Example OvidSP Search terms for Medline database**
